# Prognostic importance of frailty and multimorbidity alongside disease risk in older adults with multiple myeloma

**DOI:** 10.1093/geroni/igaf122.3646

**Published:** 2025-12-31

**Authors:** Samantha Rizzo, Hannah Tosi, Chunlei Zheng, Jennifer La, Jane Driver, Nikhil Munshi, Nathanael Fillmore, Clark DuMontier

**Affiliations:** VA Boston Healthcare System, Boston, Massachusetts, United States; VA Boston Healthcare System, Boston, Massachusetts, United States; VA Boston Healthcare System, Boston, Massachusetts, United States; VA Boston Healthcare System, Boston, Massachusetts, United States; VA Boston Healthcare System, Brockton, Massachusetts, United States; VA Boston Healthcare System, Boston, Massachusetts, United States; VA Boston Healthcare System, Boston, Massachusetts, United States; VA Boston Healthcare System, Boston, Massachusetts, United States

## Abstract

Older adults with multiple myeloma (MM) often have frailty and multiple conditions (multimorbidity) at the time of diagnosis, representing a more complex population than younger, healthier patients in clinical trials. With data from the national Veterans Affairs (VA) electronic health record and VA Cancer Registry, we used machine learning to understand the degree to which frailty (measured via the electronic VA-Frailty Index) and multimorbidity (chronic conditions from the CMS Chronic Conditions Warehouse) predict clinical outcomes alongside MM stage and other markers of disease-risk. We performed repeated cross-validation to train and test models to predict 3-year mortality, starting by only including MM-specific predictors in the first round of training-testing, followed by adding in frailty and multimorbidity predictors in the second round (holistic model). A total of 4416 patients age ≥ 65 years with newly diagnosed MM who received treatment at the VA between 2004 and 2020 were included. The holistic model showed superior predictive performance (AUROC 0.71) compared to the model that was developed using MM-specific predictors only (AUROC 0.66, t-test p < 0.001). The top predictors that contributed to mortality risk were age, hospitalization in the 180 days before starting MM treatment, diuretic use, hemoglobin < 10 g/dL, and frailty. A significant portion of each predictors’ impact on mortality was mediated through interactions with other variables. Although disease risk normally drives prognostication in MM, our findings reveal that frailty and multimorbidity significantly improve mortality prediction through both their individual contributions and synergistic interactions.

